# Bisphosphonates: are they standard of care for the treatment of breast cancer?

**DOI:** 10.1038/sj.bjc.6603836

**Published:** 2007-06-12

**Authors:** K I Pritchard

**Affiliations:** 1 Toronto-Sunnybrook Regional Cancer Centre, Clinical Trials and Epidemiology, 2075 Bayview Avenue, Toronto, Ontario, Canada M4N 3M5

In this issue, the *British Journal of Cancer* publishes an interesting and well-conducted meta-analysis of published randomised trials of the use of clodronate in breast cancer. Outcomes in the meta-analysis include overall survival, metastasis free-survival and bone metastasis-free survival. The meta-analysis is conducted independently for patients with advanced breast cancer and in the adjuvant setting.

In spite of several well-known positive studies in this area, particularly a large trial by Powles *et al* (2002, 2006) in the adjuvant setting, the current meta-analysis does not show significant benefit for the use of clodronate for any of the end points mentioned above.

In the early breast cancer setting, this seems particularly related, as shown clearly in the forest plots in Figures 1–3 of this meta-analysis, to heterogeneity among the trials. Although the largest study (Powles *et al*, 2002, 2006) shows significant improvement in overall survival and bone metastasis-free survival, as does the smaller trial by Diel *et al* (1998) the Saarto Trial (Saarto *et al*, 2001, 2004) remains an outlier, showing a significant negative effect on both overall survival and non-skeletal metastasis-free survival, and a trend towards a negative effect on bone metastasis-free survival. The Saarto Trial is in fact very different in outcome from any of the other studies in either the adjuvant setting or in metastatic disease, all of which either trend towards a positive effect or show a significant positive effect for the use of clodronate. Although the authors use correct statistical techniques (random effects model) for the meta-analysis, the ‘opposite’ outcomes of the Saarto study are problematic in interpreting a meta-analysis of this group of trials. These difficulties are compounded by the fact that most of the trials included in the meta-analysis are small. There are only slightly more than 500 patients in all of the trials in advanced breast cancer, and slightly more than 1650 in the trials conducted in the adjuvant setting. This leaves the meta-analysis relatively underpowered to demonstrate a significant effect on the outcomes being studied.

Another methodologic problem of this, as of other meta-analyses based on published data, is that there is limited ability to examine effects over time. Powles *et al* (2002, 2006) have clearly outlined in their publications that 2 years of clodronate resulted in an early effect, which tended to wane over time after it was discontinued. They have interpreted this to suggest that if clodronate had been given longer, the effects might have been more sustained, and this may well be the case. The use of hazard ratios (HR) based on 5 year overall survival, bone metastasis-free survival and non-skeletal metastasis-free survival does not provide the ability to examine trends over time, and may tend to minimize effects which may occur early because they directly relate to the ongoing utilization of the bisphosphonate. The authors accessed Kaplan–Meier curves or survival tables wherever possible, which allowed some additional information, particularly for overall survival. However, they were also reduced to using 5-year survival estimates for some trials. This not only minimized the ability of the authors to examine trends over time, but also reduced their access to accurate 5-year survival rates, which adds a further methodologic problem.

Within these methodologic limitations, however, it seems that there may be more evidence tending to support a benefit for overall survival and for bone metastasis-free survival than for non-skeletal metastasis-free survival. For non-skeletal metastasis-free survival, the HR of 0.89 (confidence intervals (CI)=0.40–1.98) for adjuvant therapy is not as ‘encouraging’ as either that for bone metastasis-free survival which is 0.77 for advanced breast cancer and 0.68 (CI=0.38–1.23) for adjuvant therapy, or for overall survival which is 0.73 (CI=0.46–1.14) in advanced breast cancer and 0.75 (CI=0.31–1.82) for adjuvant therapy.

Within the methodologic limitations described, the authors provide an excellent meta-analysis and summary of the published material available on this subject. This analysis and review is helpful in assessing the current state of the art of clodronate therapy in the adjuvant or metastatic setting. It is clear from other studies (Hortobagyi *et al*, 1996, 1998; Lipton *et al*, 1997) that bisphosphonates used in the metastatic setting prevent or delay skeletal-related events, reduce pain and improve quality of life. However, it is less clear that clodronate or any bisphosphonate reduces the development of subsequent bone metastases in bone metastasis-free patients with other metastatic disease, or in the adjuvant setting, nor that clodronate reduces bone metastases or metastases in general or improves overall survival. Thus, results from the already completed NSABP B-34 trial will be crucial in determining the standard of care in the adjuvant setting. While the data of Hortobyagi and Lipton is probably sufficient to suggest the use of bisphosphonates (Hortobagyi *et al*, 1996, 1998; Lipton *et al*, 1997) in patients who have already developed bone metastases, the current meta-analysis would suggest that there is yet insufficient data to use bisphosphonates either in the setting of metastatic disease which does not involve bone or in adjuvant therapy. While the trends from these relatively small studies even in meta-analysis do not provide clear results, the results from the large B-34 study in which 3000 patients were randomized to clodronate *vs* placebo given for 3 years should prove definitive. This trial was activated in December 2000 and completed accrual in 2004, but sufficient events have not as yet occurred to provide adequate powering for release of results and are not expected to occur until mid-2008 (E Mamounas, personal communication, February 2007).

It is particularly important that we wait for definitive results from the large trials or from individual patient meta-analysis before adopting the routine use of bisphosphonates in the adjuvant or the metastatic setting without bone involvement. Currently, we are just beginning to understand the magnitude of a relatively newly reported complication of bisphosphonates, that of jaw osteonecrosis. While this has been reported much more commonly in relation to long-term administration of intravenous bisphosphonates (Conte *et al*, 1996; Bamias *et al*, 2005; Conte and Guarneri, 2005; Bilezikian, 2006; Wendler and Shalowitz, 2006; Woo *et al*, 2006) occasional reports have occurred with oral drugs such as clodronate (Mavrokokki *et al*, 2007), alendronate (Fosamax) (Ruggiero *et al*, 2004; American Dental Association, 2006; Mavrokokki *et al*, 2007) and risedronate (Actonel) (Ruggiero *et al*, 2004; Mavrokokki *et al*, 2007). It will be important to have results from a large randomized trial such as B-34, which will tell us not only the efficacy of clodronate in the adjuvant setting but also the relative risk of side effects in comparison to a placebo control arm.

In the meantime, the use of bisphosphonates in the adjuvant setting remains appropriate only when given as suggested in guidelines (Brown and Josse, 2002; Hillner *et al*, 2003) for osteoporosis, or in the setting of clinical trials. Their use to reduce skeletal or non-skeletal-related metastases or to improve overall survival is not supported by currently available data.
